# Editorial: Community series in novel antipsychotics within and beyond clinical trials: symptom-based treatment of psychiatric disorders with D3-D2 partial agonists, volume II

**DOI:** 10.3389/fpsyt.2023.1266566

**Published:** 2023-08-21

**Authors:** Peter Falkai, Zsófia Borbála Dombi

**Affiliations:** ^1^Department of Psychiatry and Psychotherapy, University Hospital, Ludwig Maximilian University of Munich, Munich, Germany; ^2^Global Medical Division, Gedeon Richter Plc., Budapest, Hungary; ^3^Department of Psychiatry, University of Oxford, Oxford, United Kingdom

**Keywords:** partial agonists, dopamine, symptom-based treatment, real-world evidence, antipsychotic

Psychiatric disorders are a heterogenous group of conditions characterized by various symptoms and complex underlying pathological mechanisms (1). Aiming to simplify diagnostic and treatment strategies, current definitions of psychiatric disorders are rather categorical, thereby overlooking their inherent complexity (2). To give an example, according to the Diagnostic and Statistical Manual of Mental Disorders, Fifth Edition (DSM-5) (3), schizophrenia is diagnosed based on the presence of two symptoms out of five, meaning that two patients with the same diagnosis can exhibit a completely different symptom profile (2). This leads to the question: how should we treat patients with the same diagnosis but different symptomatology? A solution for this puzzle is a symptom-based treatment which refers to the notion that psychiatrists make their treatment decisions primarily based on the actual symptoms the patient experiences rather than the diagnosis itself (4). This transdiagnostic approach helps to make treatment decisions that are more personalized and therefore might have an increased likelihood of being effective (4).

When it comes to the treatment of psychiatric disorders, the dopaminergic system becomes a key target, as its dysregulation has a major role in the pathophysiology of a wide range of symptoms that are present across various psychiatric disorders, like schizophrenia or bipolar disorder (5). Antipsychotics, a class of drugs that predominantly target dopamine receptors, are key medications utilized in the treatment of these conditions. While dopamine D2 receptors are associated with positive, psychotic, or manic symptoms (6, 7), D3 receptors are thought to be related to negative and depressive symptomatology (8, 9). Partial agonists, a new class of antipsychotic drugs, have the ability to target these receptors according to the actual needs of the patients—they are smart drugs that exhibit agonistic or antagonistic effects depending on the need and the dopamine concentration present (10, 11). The seven articles of the present Research Topic, therefore, aimed to explore how symptoms of psychiatric disorders can be addressed with dopamine D3-D2 partial agonists.

Acute schizophrenia patients are often hospitalized due to the intensity of their positive symptoms such as hallucinations, delusions, or paranoia (12). These patients can also exhibit hostile behavior which further increases the necessity of being hospitalized (13, 14). The primary treatment goal here is to decrease the psychotic symptomatology and hostility so that they can continue their treatment as outpatients and be integrated back into society (12). Usually, these patients need higher doses of antipsychotics (15) or combination treatment (16) to stabilize, which in the long run can be reduced or fine-tuned as needed. A study by Galmes and Rancans involving 14 patients with acute symptoms of schizophrenia and schizoaffective disorder examined the efficacy of high-dose cariprazine in this patient population. The approved dose range of cariprazine is between 1.5 and 6.0 mg/day; however, in the regulatory clinical trials, higher doses were also examined. Patients achieved significant improvement in positive, negative, and hostile symptoms as measured by the Brief Psychiatric Rating Scale (BPRS) over a 2-week hospitalized period with 6.0, 7.5, or 9.0 mg cariprazine per day. Higher doses may be associated with a higher probability of adverse events; however, in this study, only one patient developed severe akathisia and therefore terminated the treatment.

Another core symptom of schizophrenia is the negative symptom domain which is composed of five constructs: anhedonia, avolition, alogia, asociality, and blunted affect (17). Negative-symptom patients can respond remarkably to medications targeting the D3 receptors (18), which has been showcased by three articles in the Research Topic authored by Pappa et al., Ivanova et al., and Vrublevska. Pappa et al. where a 6-month pilot study to evaluate the effectiveness of cariprazine in early psychosis patients (*n* = 10) with prominent or predominant negative symptoms was conducted. The seven patients who completed the study achieved significant improvement in their negative symptomatology; they had a 53% mean score reduction in the Positive and Negative Syndrome Scale (PANSS) negative subscale. Similarly, Ivanova et al. found cariprazine to be an effective and well-tolerated medication in two patients with early-onset schizophrenia who had predominant negative symptoms. Following these patients for 18 months, the authors reported a 29.8% improvement on the PANSS negative subscale. Finally, Vrublevska published a case report describing a young but severely ill patient who had recurrent relapses of schizophrenia with persistent negative symptoms. Several trials of different antipsychotic combinations were tried without sufficient response and with extrapyramidal side effects. After initiating combination treatment with cariprazine and olanzapine, all symptoms as well as the overall functioning improved. A similar case was described by Schölin et al., whereby a schizophrenia patient with a pronounced functional deficit was successfully treated with cariprazine. The patient had experienced a wide range of symptoms with increasing severity as well as a history of drug abuse. After no success in treating his negative symptoms with first olanzapine and then risperidone, a switch to cariprazine was decided, leading to marked functional improvement and no psychotic symptoms after 6 months of discharge. Although the described patient was not treatment-resistant, this case highlights the difficulty in finding the right treatment strategy for patients with residual schizophrenia symptoms.

Addressing the Research Topic of non-responsivity, Vasiliu (a) reviewed the available evidence regarding the efficacy of third-generation antipsychotics in combination with clozapine in patients with treatment-resistant schizophrenia. Combinations with aripiprazole and cariprazine are most supported and can positively impact not only the positive, negative, and general symptoms but the metabolic profile too. Last, but not least, Vasiliu (b) also submitted a scoping review focusing on the pharmacogenetics of new-generation antipsychotics. The author highlighted why exploring the relationship between clinical efficacy and gene variations is an important aspect of personalized medicine and detailed the available evidence regarding aripiprazole, brexpiprazole, cariprazine, lumateperone, and pimavanserin. One of the main take-away messages of the review is that determining the CYP2D6 metabolizer status can aid the better administration of aripiprazole and brexpiprazole and the CYP3A4 status is relevant for cariprazine, lumateperone, and pimavanserin.

Overall, the present Research Topic underlines how novel antipsychotics offer the opportunity for a more personalized, patient-centered treatment.

## Author contributions

PF: Conceptualization, Supervision, Writing—review and editing. ZBD: Data curation, Writing—original draft.
